# Tandem affinity purification protocol for isolation of protein complexes from *Schizosaccharomyces pombe*

**DOI:** 10.1016/j.xpro.2022.101137

**Published:** 2022-01-28

**Authors:** Lubos Cipak, Tomas Selicky, Jan Jurcik, Ingrid Cipakova, Michaela Osadska, Veronika Lukacova, Peter Barath, Juraj Gregan

**Affiliations:** 1 Department of Genetics, Cancer Research Institute, Biomedical Research Center of the Slovak Academy of Sciences, Dubravska Cesta 9, 845 05 Bratislava, Slovakia; 2 Medirex Group Academy, Novozamocka 67, 949 05 Nitra, Slovakia; 3 Department of Glycobiology, Slovak Academy of Sciences, Institute of Chemistry, Dubravska Cesta 9, 845 05 Bratislava, Slovakia; 4 Department of Applied Genetics and Cell Biology, Institute of Microbial Genetics, University of Natural Resources and Life Sciences, Vienna (BOKU), Konrad Lorenz Strasse 24, 3430 Tulln an der Donau, Austria

**Keywords:** Cell Biology, Model Organisms, Protein Biochemistry, Protein expression and purification

## Abstract

Many cellular processes require the activities of complex molecular machines composed of several protein subunits. Insights into these systems can be gained by isolation of protein complexes followed by *in vitro* analyses determining the identity, posttranslational modifications, and interactions among proteins. Here, we present a protocol for tandem affinity purification (TAP) of protein complexes from the fission yeast *Schizosaccharomyces pombe*. The protocol employs cells expressing C-terminally TAP-tagged proteins and is suitable for the analysis of purified proteins by mass spectrometry.

For complete information on the use and execution of this protocol, please refer to Cipakova et al. (2019).

## Highlights

• Step-by-step protocol for purification of *S. pombe* proteins under native conditions
• Purification of Ntr1, Ntr2, and Brr2 together with co-purifying proteins
• The protocol has been validated for various *S. pombe* proteins

## Graphical abstract

**Figure:**
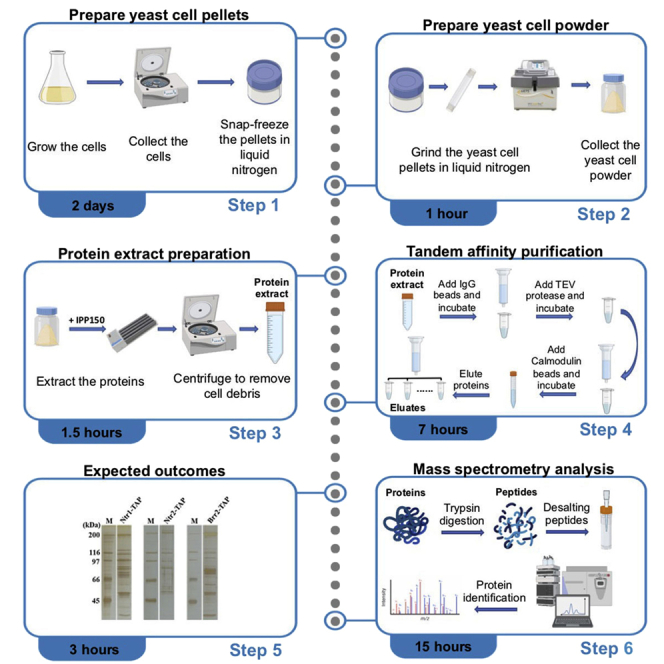

## Before you begin

Tandem affinity purification (TAP) protocol has been developed to purify protein complexes from the budding yeast *Saccharomyces cerevisiae* (Rigaut et al., 1999). We have adapted this protocol for the fission yeast *Schizosaccharomyces pombe* and used it to isolate various TAP-tagged proteins, including protein kinases (Cipak et al., 2009, 2013), cohesin subunits (Phadnis et al., 2015; Sanyal et al., 2018), monopolin proteins (Rumpf et al., 2010), recombination proteins (Cipak et al., 2009; Sevcovicova et al., 2021) and spliceosomal factors (Aronica et al., 2016; Cipakova et al., 2019; Mikolaskova et al., 2021)*.*

**Figure 1:**
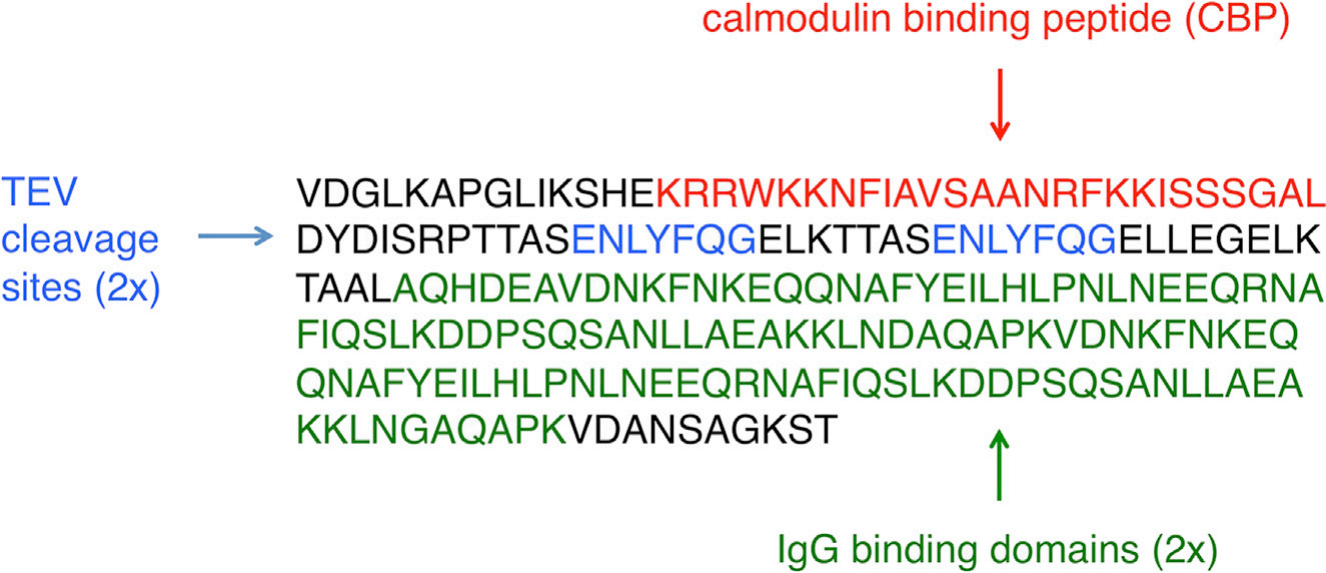
Schematic representation and amino acid sequence of the TAP tag

The TAP tag used in this protocol consists of a calmodulin-binding peptide and two IgG-binding domains of protein A, separated by a tobacco etch virus (TEV) protease cleavage site (calmodulin-binding peptide−TEV−protein A) (Figure 1). To generate *S. pombe* cells expressing C-terminally TAP-tagged proteins, refer to our previous work (Cipak et al., 2009).

Protocols optimized for TAP purification of other *S. pombe* protein complexes have been previously published (Gerace and Moazed, 2015; Gould et al., 2004). In addition to calmodulin-binding peptide−TEV−protein A tag, various combinations of tags such as 3xFLAG−TEV−protein A (Zilio and Boddy, 2017) or protein G−TEV−streptavidin-binding peptide (Goodfellow and Bailey, 2014) can be used for TAP-tagging and purification. The basic principles of these protocols are similar and our protocol contains only minor modifications. One such modification is using a Freezer/Mill grinder cooled with liquid nitrogen to prepare cryomilled yeast cell powder for producing protein extracts. The Freezer/Mill is very efficient in breaking *S. pombe* cells and minimizes protein degradation.

Prior to the protein extraction and affinity purification, prepare all the buffers and store them at 4°C. Protease and phosphatase inhibitors should be added immediately before the buffers are used. All steps need to be performed on ice in a cold room (4°C) or using refrigerated boxes and centrifuges, unless stated otherwise. To prevent contamination of reagents, plastic material and samples, always wear clean lab coat and powder-free gloves.

### Prepare yeast cell pellets

**Timing: 2 days**

This section details the collection of yeast cells and preparation of yeast cell powder from mitotic cultures. Although we describe here how to prepare the yeast cell powder from asynchronous mitotic cells, this procedure can be adapted for preparation of cell powder from synchronized or meiotic cultures.

1. Inoculate *S. pombe* cells expressing the TAP-tagged protein of interest and the untagged control *S. pombe* cells in YE+5S medium in separate baffled flasks at the OD_600_=0.1.
2. Grow the cells at 32°C with gentle shaking (∼150 rpm) until they reach stationary phase (OD_600_>1.5). This takes about 8–10 h.

**CRITICAL:** It is important to dilute *S. pombe* cells immediately after they reach stationary phase. Keeping cells for a long time in stationary phase makes it difficult for them to recover after dilution in fresh medium.

**Alternative:** In case there is a need to isolate proteins from synchronized or meiotic cultures, various synthetic media can be used. Recipes for YE+5S and other yeast media are described in (Cipak et al., 2014; Huraiova et al., 2020; Sabatinos and Forsburg, 2010).

***Note:*** YE+5S medium contains: 5 g/L yeast extract, 30 g/L glucose, 0.15 g/L adenine, 0.1 g/L uracil, 0.1 g/L L-histidine, 0.1 g/L L-lysine and 0.1 g/L L-leucine.

3. Next day, dilute the cells in fresh YE+5S medium (6 L, see the Note below) to the OD_600_=0.1 and incubate until the OD_600_=0.8–1.0 (∼8–10 h).
4. Cool the cultures in ice-cold water bath (∼10 min).
5. Collect the cells by centrifugation at 3,000 g for 5 min at 4°C and remove the medium.
6. Wash the collected cells with 100 mL of ice-cold ddH_2_O by gently suspending the pellet.

**CRITICAL:** Do not vortex the cells during resuspension and washing steps, use clean and sterile plastic spoon instead to release them into solution.

7. Centrifuge the cells at 3,000 g for 5 min at 4°C and immediately remove the supernatant.
8. Snap-freeze the pelleted cells in liquid nitrogen.

**Pause point:** Frozen yeast pellets can be stored at −80°C for several weeks.

***Note:*** Untagged *S. pombe* cells isogenic to the strain expressing the TAP-tagged protein, referred to as untagged control cells, should be grown in parallel and used as negative control (mock sample). This control serves various purposes, such as identification of unspecific protein contaminants. Strains expressing empty TAP tag or unrelated TAP-tagged protein can be used as additional negative controls. It is also useful to use a strain expressing TAP-tagged protein that has been previously successfully purified as a positive control. Additionally, yeast cell pellets should be prepared for at least two biological replicates per condition to ensure reproducibility of results.

***Note:*** The amount of yeast cell culture might be scaled up depending on the expression level of the TAP-tagged protein or the experimental setup (e.g., collection of cells at multiple time points). In our experience, 6–8 L of single time point yeast cell culture gives the optimal amount of biological material (30–40 g of wet cell pellet) for its further processing. The usual yield is about 4–5 g of wet yeast cell pellet from 1 L of yeast cell culture of OD_600_=0.8–1.0.

### Prepare yeast cell powder

**Timing: 1 h**

9. Transfer the frozen yeast cell pellet from −80°C into liquid nitrogen. Cool the grinding vial in liquid nitrogen and fill it with the yeast cell pellet. Keep the filled vial in the liquid nitrogen until the grinding step.
10. Place the grinding vial containing the yeast pellet into the Freezer/Mill cryogenic grinder cooled with liquid nitrogen. Set up the program (7 cycles, pre-cool 10 min, run 3 min, cool 2 min, rate 15 CPS) and follow the operation manual to start the grinding.
11. Collect the yeast cell powder from the grinding vial into clean tubes cooled in liquid nitrogen.

**Pause point:** Yeast cell powder can be stored at −80°C for several weeks.

**CRITICAL:** Do not allow the yeast cell pellet or yeast cell powder to thaw during manipulation. It is critical to keep the sample frozen. This makes the sample “grindable” and prevents its alterations and protein degradation.

***Note:*** Liquid nitrogen can be hazardous. When working with liquid nitrogen, always wear cryogenic gloves to protect your hands and safety goggles/shield to protect your eyes/face. Do not splash liquid nitrogen on clothes or unprotected skin.

## Key resources table

**Table:** 

REAGENT or RESOURCE	SOURCE	IDENTIFIER
**Antibodies**		

Rabbit antiperoxidase antibody linked to peroxidase (PAP1)	Dako	N/A

**Chemicals, peptides, and recombinant proteins**		

Tris-HCl (pH 8.0)	Thermo Fisher Scientific	Cat#: 15568025
Sodium chloride (NaCl)	Sigma-Aldrich	Cat#: S3014
Glycerol	Sigma-Aldrich	Cat#: G5516
IGEPAL CA-630 (NP-40)	Sigma-Aldrich	Cat#: I8896
Phenylmethanesulfonyl fluoride (PMSF)	Sigma-Aldrich	Cat#: P7626
Ethylenediaminetetraacetic acid solution (EDTA)	Sigma-Aldrich	Cat#: 03690
D,L-Dithiothreitol (DTT)	Sigma-Aldrich	Cat#: D0632
Magnesium acetate tetrahydrate	Sigma-Aldrich	Cat#: M5661
Imidazole	Sigma-Aldrich	Cat#: 68268
Calcium chloride dihydrate (CaCl_2_)	Merck	Cat#: 102382
2-Mercaptoethanol	Sigma-Aldrich	Cat#: M3148
Ethylene glycol-bis(2-aminoethyl ether)-N,N,N,N-tetra acetic acid (EGTA)	Sigma-Aldrich	Cat#: 03777
Sodium fluoride (NaF)	Sigma-Aldrich	Cat#: 201154
β-Glycerol phosphate disodium salt pentahydrate	Sigma-Aldrich	Cat#: 50020
Sodium pyrophosphate decahydrate	Sigma-Aldrich	Cat#: 221368
Sodium orthovanadate (Na_3_VO_4_)	Sigma-Aldrich	Cat#: 450243
cOmplete EDTA-free protease inhibitor cocktail	Roche	Cat#: COEDTAF-RO
PhosSTOP	Sigma-Aldrich	Cat#: 4906845001
IgG Sepharose^TM^ 6 Fast Flow	GE Healthcare	Cat#. 17-0969-01
Calmodulin Sepharose^TM^ 4B	GE Healthcare	Cat#. 17-0529-01
AcTEV protease	Invitrogen	Cat#: 12575023
Benzonase Nuclease	Merck	Cat#: 70664
Iodoacetamide	Sigma-Aldrich	Cat#: 1149
Trifluoroacetic acid	Sigma-Aldrich	Cat#: 80457
Acetonitrile	Merck	Cat#: 1000291000
Sequencing Grade Modified Trypsin	Promega	Cat#: V5111
LiChroprep RP-18	Merck	Cat#: 1093030100

**Experimental models: Organisms/strains**		

*S. pombe*: Strain background: *h*^*-*^*ORF-TAP::KanMX6*	N/A	N/A

**Other**		

Refrigerated Incubator with built-in Shaker	N-BIOTEK	Cat#: NB-250VQ
Refrigerated Large-volume Centrifuge	N/A	N/A
Large Capacity Cryogenic Grinder	SPEX SamplePrep	Cat#: 6875
Small Grinding Vial Set	SPEX SamplePrep	Cat#: 6751
Mid-Size Grinding Vial Set	SPEX SamplePrep	Cat#: 6881
Large Grinding Vial Set	SPEX SamplePrep	Cat#: 6801
MSE Soniprep 150 Ultrasonic Disintegrator	MSE	Cat#: JM458
Refrigerated High-speed Centrifuge Z 36 HK	Hermle	Cat#: 302.00 V03
Platform rotator	IKA	Cat#: 0004011000
Rotatory wheel	IKA	Cat#: 0004015000
Poly-Prep chromatography column (2 mL bed volume)	Bio-Rad	Cat#: 7311550
Econo-Pac chromatography column (20 mL bed volume)	Bio-Rad	Cat#: 7321010
Refrigerated Centrifuge	Eppendorf	Cat#: 5702 R
Refrigerated Microcentrifuge	Eppendorf	Cat#: 5418 R
Mini-PROTEAN Tetra Cell	Bio-Rad	Cat#: 1658000EDU
2-Gel Tetra and Blotting Module	Bio-Rad	Cat#: 1660827EDU
Protein LoBind Tube	Eppendorf	Cat#: 022431081
Vacuum Concentrator plus	Eppendorf	Cat#: 5305000304

The following reagents should be stored at 4°C: Phenylmethanesulfonyl fluoride (PMSF), Imidazole.

The following reagents should be stored at −20°C: cOmplete EDTA-free protease inhibitor cocktail, AcTEV protease, Benzonase Nuclease, D,L-Dithiothreitol (DTT).

## Materials and equipment

The following reagents can be prepared ahead of time. The indicated pH is measured at room temperature (20°C–25°C).

### 1M Tris-HCl (pH 8.0)

Dissolve 12.11 g of Tris base in 80 mL of ddH_2_O. Adjust the pH to 8.0 by adding concentrated HCl. Filter sterilize (0.22 μm) and store at 25°C protected from light.

### 5M NaCl

Dissolve 29.22 g of NaCl in 80 mL of ddH_2_O. Adjust the volume to 100 mL with ddH_2_O. Sterilize by autoclaving and store at 25°C.

### 50% Glycerol

Dilute 250 mL of 100% glycerol with 250 mL of ddH_2_O. Filter sterilize (0.22 μm) and store at 25°C.

### 10% NP-40

Dilute 5 mL of 100% NP-40 in 45 mL of ddH_2_O. Store at 25°C.

### 100 mM PMSF

Dissolve 8.71 g of PMSF in 50 mL of 100% isopropanol. Store at −20°C for up to 6 months.

### 1M Sodium fluoride

Dissolve 2.10 g of NaF in 50 mL of ddH_2_O. Filter sterilize (0.22 μm) and store at −20°C.

### 100 mM Sodium pyrophosphate

Dissolve 2.23 g of sodium pyrophosphate decahydrate in 50 mL of ddH_2_O. Filter sterilize (0.22 μm) and store at −20°C.

### 1M β-Glycerol phosphate

Dissolve 6.12 g of β-Glycerol phosphate disodium salt pentahydrate in 20 mL of ddH_2_O. Filter sterilize (0.22 μm) and store at −20°C.

### 200 mM Sodium orthovanadate (pH 10.0)

Dissolve 3.68 mg of Na_3_VO_4_ in 9 mL of ddH_2_O. Adjust pH to 10.0 by HCl (solution will turn yellow). Heat the solution to 95°C for 5 min (solution will become colorless). Adjust the volume to 10 mL with ddH_2_O. Cool on ice, aliquot and store at −20°C (can be kept up to 2 months).

### 1M DTT

Dissolve 308.5 mg of DTT in 2 mL of ddH_2_O and sterilize through a 0.22 μm filter. Make 0.1 mL aliquots and store at −20°C (can be kept up to 6 months).

### 1M Magnesium acetate

Dissolve 10.72 g of magnesium acetate tetrahydrate in 50 mL of ddH_2_O. Filter sterilize (0.22 μm) and store at 25°C.

### 1M CaCl_2_

Dissolve 7.35 g of calcium chloride dihydrate in 50 mL of ddH_2_O. Filter sterilize (0.22 μm) and store at 25°C.

### 100 mM EGTA (pH 8.0)

Add 760.7 mg of EGTA to about 15 mL of ddH_2_O. Bring pH to 11.0 with 1 M NaOH. Then adjust pH to 8.0 with HCl and add ddH_2_O to a final volume of 20 mL. Filter sterilize (0.22 μm) and store at 25°C.

### 500 mM Iodoacetamide

Dissolve 46 mg of iodoacetamide in 0.5 mL ddH_2_O. Prepare immediately before use and keep out of light. Store at 25°C.

### 10% Trifluoroacetic acid

Add 1 mL of 100% trifluoroacetic acid into 9 mL ddH_2_O. Store at 25°C.

### 50% C18 resin slurry

Transfer approximately 2 mL of the C18 resin LiChroprep RP-18 into graduated 15 mL screw-cap tube. Add equal volume of acetonitrile. Store at 25°C.

### Sequencing trypsin solution

Reconstitute a vial containing 20 μg of Sequencing Grade Modified Trypsin in 0.16 mL of supplied Trypsin Resuspension Buffer (50 mM acetic acid). Mix well, prepare 10 μL aliquots and store at −20°C.

**CRITICAL:** To avoid inhalation of PMSF, NaF, Na_3_VO_4_, DTT, iodoacetamide, trifluoroacetic acid, acetonitrile, LiChroprep RP-18 and 2-Mercaptoethanol, handle these reagents in a chemical fume hood while wearing appropriate personal protective equipment, such as lab coat, gloves and safety goggles.

**Table:** IPP150 buffer

Reagent	Final concentration	Amount
Tris-HCl (pH 8.0) (1 M)	50 mM	5 mL
NaCl (5 M)	150 mM	3 mL
Glycerol (50%)	10%	20 mL
NP-40 (10%)	0.1%	1 mL
PMSF (100 mM)	1 mM	1 mL
NaF (1 M)	20 mM	2 mL
Sodium pyrophosphate (100 mM)	5 mM	5 mL
β-Glycerol phosphate (1 M)	10 mM	1 mL
Na_3_VO_4_ (200 mM)	1 mM	0.5 mL
cOmplete EDTA-free tablet	n/a	2 tablets
ddH_2_O	n/a	61.5 mL
**Total**	**n/a**	**100 mL**

Prepare on the day of the experiment, filter sterilize and keep at 4°C. Adjusting the pH after mixing all the ingredients is not needed.

**CRITICAL:** The PMSF, NaF, sodium pyrophosphate, β-Glycerol phosphate, Na_3_VO_4_, and cOmplete EDTA-free protease inhibitors should be added into IPP150 buffer immediately prior to use.

**Table:** TEV cleavage buffer (TCB)

Reagent	Final concentration	Amount
Tris-HCl (pH 8.0) (1 M)	10 mM	0.5 mL
NaCl (5 M)	150 mM	1.5 mL
EDTA (500 mM)	0.5 mM	0.05 mL
Glycerol (50%)	10%	10 mL
NP-40 (10%)	0.1%	0.5 mL
DTT (1 M)	1 mM	0.05 mL
PMSF (100 mM)	1 mM	0.5 mL
cOmplete EDTA-free tablet	n/a	1 tablet
PhosSTOP	n/a	5 tablets
ddH_2_O	n/a	36.9 mL
**Total**	**n/a**	**50 mL**

Prepare on the day of the experiment, filter sterilize and keep at 4°C. Adjusting the pH after mixing all the ingredients is not needed.

**CRITICAL:** The DTT, PMSF, PhosSTOP, and cOmplete EDTA-free protease inhibitors should be added into TCB immediately prior to use.

**Table:** Calmodulin binding buffer 1 (CBB1)

Reagent	Final concentration	Amount
Tris-HCl (pH 8.0) (1 M)	10 mM	0.5 mL
NaCl (5 M)	150 mM	1.5 mL
Magnesium acetate (pH 7.4) (1 M)	1 mM	0.05 mL
Imidazole (pH 9.0) (1 M)	1 mM	0.05 mL
CaCl_2_ (1 M)	2 mM	0.1 mL
Glycerol (50%)	10%	10 mL
2-Mercaptoethanol	10 mM	0.035 mL
NP-40 (10%)	0.1%	0.5 mL
ddH_2_O	n/a	37.265 mL
**Total**	**n/a**	**50 mL**

Prepare on the day of the experiment, filter sterilize and keep at 4°C. Adjusting the pH after mixing all the ingredients is not needed.

**CRITICAL:** The 2-Mercaptoethanol should be added into CBB1 immediately prior to use.

**Table:** Calmodulin binding buffer 2 (CBB2)

Reagent	Final concentration	Amount
Tris-HCl (pH 8.0) (1 M)	10 mM	0.5 mL
NaCl (5 M)	150 mM	1.5 mL
Magnesium acetate (pH 7.4) (1 M)	1 mM	0.05 mL
CaCl_2_ (1 M)	2 mM	0.1 mL
2-Mercaptoethanol	1 mM	0.0035 mL
ddH_2_O	n/a	47.8465 mL
**Total**	**n/a**	**50 mL**

Prepare on the day of the experiment, filter sterilize and keep at 4°C. Adjusting the pH after mixing all the ingredients is not needed.

**CRITICAL:** The 2-Mercaptoethanol should be added into CBB2 immediately prior to use.

**Table:** Elution buffer (EB)

Reagent	Final concentration	Amount
Tris-HCl (pH 8.0) (1 M)	10 mM	0.1 mL
NaCl (5 M)	150 mM	0.3 mL
Magnesium acetate (pH 7.4) (1 M)	1 mM	0.01 mL
EGTA (100 mM)	2 mM	0.2 mL
2-Mercaptoethanol	1 mM	0.0007 mL
ddH_2_O	n/a	9.3893 mL
**Total**	**n/a**	**10 mL**

Prepare on the day of the experiment, filter sterilize and keep at 4°C. Adjusting the pH after mixing all the ingredients is not needed.

**CRITICAL:** The 2-Mercaptoethanol should be added into EB immediately prior to use.

**Table:** 50 mM Phosphatase inhibitors mix (50×)

Reagent	Final concentration	Amount
NaF (1 M)	50 mM	0.05 mL
Sodium pyrophosphate (100 mM)	50 mM	0.5 mL
β-Glycerol phosphate (1 M)	50 mM	0.05 mL
Na_3_VO_4_ (200 mM)	50 mM	0.25 mL
ddH_2_O	n/a	0.15 mL
**Total**	**n/a**	**1 mL**

Prepare 0.1 mL aliquots and store at −20°C.

**Table:** 0.1% TFA

Reagent	Final concentration	Amount
Trifluoroacetic acid (10%)	0.1%	1 mL
ddH_2_O	n/a	9 mL
**Total**	**n/a**	**10 mL**

Prepare and store at 25°C.

**Table:** TA70

Reagent	Final concentration	Amount
Trifluoroacetic acid (0.1%)	0.03%	3 mL
Acetonitrile (100%)	70%	7 mL
**Total**	**n/a**	**10 mL**

Prepare and store at 25°C.

## Step-by-step method details

### Protein extract preparation

**Timing: 1.5 h**

This section details step-by-step procedure of the protein extract preparation.

1. Transfer the yeast cell powder into appropriate tubes. Thaw the yeast cell powder on ice (∼2 min) and resuspend it in the ice-cold IPP150 buffer.

***Note:*** Use 3 mL of ice-cold IPP150 buffer per 1 g of the yeast cell powder.

**CRITICAL:** Protease and phosphatase inhibitors are critical for the stability of proteins and their post-translational modifications during the extraction step. In our experience, the lack of protease and phosphatase inhibitors in extraction of the IPP150 buffer leads to significant degradation of proteins and loss of their post-translational modifications and might prevent successful isolation of native protein complexes.

2. To extract the proteins, gently rotate the tubes with lysates on a rotatory wheel in the cold room (4°C) for 30 min.

***Optional:*** To generate homogenous lysates, the samples can be sonicated. The sonication conditions need to be adjusted to reduce the generation of foam and splashes. Always keep the tubes with lysates on ice. Note that some protein complexes are sensitive to sonication.

We homogenize yeast cell powder resuspended in IPP150 buffer in 300 mL centrifuge tube, kept on ice, using MSE Soniprep 150 set at 6 amplitude microns (5 cycles of 10 s and 30 s cooling periods in between cycles).

***Optional:*** In case the isolated TAP-tagged protein is bound to chromatin, the IPP150 buffer can be supplemented with Benzonase. Benzonase is a nuclease used for digesting DNA and releasing nuclear proteins associated with DNA. Typically, we use the ice-cold IPP150 buffer with Benzonase (20 U/mL) and Mg^2+^ ions (MgCl_2_, 1 mM) to activate Benzonase.

3. Transfer the lysate to clean centrifuge tubes. To remove cell debris, centrifuge the tubes at 40,000 g for 10 min at 4°C. Transfer the supernatant into a clean centrifuge tube and repeat the centrifugation step three more times (40,000 g, 10 min, 4°C).

**CRITICAL:** It is difficult to remove all of the supernatant containing the extracted proteins without disturbing the pellet. We advise to use pre-cooled large volume pipet to transfer the protein extract into fresh and clean centrifuge tubes.

4. Transfer the clean protein extract into 50 mL Falcon tubes.

***Optional:*** To remove remaining cell debris, the protein extract can be filtered (0.22 μm) before freezing in liquid nitrogen. Note that some proteins may remain bound to the filter.

***Optional:*** Transfer 50 μL of protein extract in an ice-cold 1.5 mL tube and store at −20°C. It can be used later to analyze the expression and stability of TAP-tagged protein.

**CRITICAL:** The stability of proteins in a crude extract is time sensitive, hence it is important to complete the protein extraction in a timely manner.

5. Proceed to the affinity purification steps or snap-freeze the protein extract in liquid nitrogen.

**Pause point:** Frozen protein extracts can be stored at −80°C for up to one month. Note that some protein complexes are sensitive to freezing and thawing.

### Tandem affinity purification

**Timing: 7 h**

These steps describe tandem affinity purification of TAP-tagged proteins together with co-purifying proteins.

6. Take 500 μL of IgG beads and mix with 10 mL of ice-cold ddH_2_O (this amount of beads is sufficient for one sample) in 15 mL Falcon tube. Gently spin the beads (400 g, 4°C, 1 min) and remove the supernatant. Wash the IgG beads with 5 mL of ice-cold IPP150 buffer (without protease and phosphatase inhibitors added). Spin again at 400 g at 4°C for 1 min. Remove the supernatant.

***Note:*** Never resuspend the IgG beads by vortexing. Resuspend the IgG beads by gently pipetting instead.

**CRITICAL:** Do not use more than 500 μL of IgG beads for one protein purification. The IgG beads have a high binding capacity, and increasing the amount of IgG beads does not necessarily result in more TAP-tagged protein binding, but it may lead to increased unspecific protein binding.

***Note:*** The IgG Sepharose^TM^ 6 Fast Flow binds ∼3 mg protein A/ml and has working interval over a pH range of 3.0–10.0.

7. Resuspend the IgG beads in 2 mL of ice-cold IPP150 buffer and mix with the protein extract. Gently incubate the sample on rotatory wheel at 4°C for 2 h to allow proteins bind to IgG beads.
8. Load the IgG beads with bound proteins onto an empty Econo-Pac chromatography column and drain by gravity flow. Wash the beads with 20 volumes (10 mL) of ice-cold IPP150 buffer and with 5 volumes (2.5 mL) of ice-cold TCB.

***Note:*** Do not forget to wash the Econo-Pac chromatography column with ice-cold ddH_2_O and ice-cold IPP150 buffer before use.

***Note:*** Make sure the IgG beads with bound proteins do not dry during the collection and washing steps.

9. Transfer the IgG beads with bound proteins into clean 2 mL Eppendorf tube. Adjust the volume to 1 mL with TCB and add additional 1 mL of TCB supplemented with 400 U of TEV protease.
10. Gently incubate the sample on rotatory wheel at 16°C for 2 h to allow the TEV protease to cleave the TAP-tag, thus releasing the proteins of interest from IgG beads.
11. Load the cleaved sample onto an empty Poly-Prep chromatography column and collect the flow-through fraction by gravity flow. After collecting the cleaved sample, gently overlay the beads with 200 μL of TCB and collect the flow-through by gravity flow. Combine the flow-throughs and adjust the volume of sample to 2 mL with TCB.

***Note:*** Do not forget to wash the Poly-Prep chromatography column with ice-cold ddH_2_O and ice-cold TCB before use.

12. Mix 2 mL of flow-through fraction and 6 μL of 1 M CaCl_2_ to titrate the EDTA present in TCB.
13. Take 100–150 μL of calmodulin beads (this amount of beads is sufficient for one sample) and mix with 10 mL of ice-cold ddH_2_O in 15 mL Falcon tube. Gently spin (800 g, 4°C, 1 min) and wash the beads with 5 mL of ice-cold CBB1. Spin again at 800 g at 4°C for 1 min and remove the supernatant.

***Note:*** The Calmodulin Sepharose^TM^ 4B has ligand density of 0.9–1.3 mg/ml and working interval over a pH range of 4.0–9.0.

14. Resuspend the calmodulin beads in 6 mL of CBB1 and mix with flow-through fraction from step 12 in 15 mL Falcon tube.
15. Gently incubate on rotatory wheel at 4°C for 2 h.
16. Load the calmodulin beads with bound proteins onto the empty Poly-Prep chromatography column and collect them by gravity flow.

***Note:*** Do not forget to wash the Poly-Prep chromatography column with ice-cold ddH_2_O and ice-cold CBB1 before use.

17. Wash the calmodulin beads with bound proteins with 10 volumes (1–1.5 mL) of ice-cold CBB1 and 5 volumes (0.5–0.75 mL) of ice-cold CBB2.

***Note:*** Make sure the calmodulin beads with bound proteins do not dry during the collection and washing steps.

18. Elute the proteins bound to Calmodulin beads using 100–150 μL of EB. After adding the EB wait for 3–5 min before collecting the eluate. Collect 8–10 eluate fractions into Protein LoBind Eppendorf tubes.

***Note:*** Prior to further analyses (e.g., mass spectrometry), the purity and complexity of eluted fractions should be checked by separating the proteins using SDS-PAGE electrophoresis followed by silver staining (Rabilloud et al., 1988). Usually, we analyze 10% of each eluate fraction to find the peak fractions.

**Pause point:** Eluates can be stored at −80°C for several weeks.

### Preparation for mass spectrometry analysis

***Note:*** Steps 19–40 are optional and should be used only if mass spectrometry analysis of purified proteins is needed.

### Protein digestion

**Timing: 12 h**

19. Add 0.5 μL 1 M DTT (5 mM) to 100 μL of eluted fraction of interest. Incubate for 30 min at 60°C to reduce the protein disulfide bonds.

***Note:*** In case the yield of eluted proteins is low, pool several eluate fractions and analyze them as a single sample. Increase the amount of reagents accordingly, except for the trypsin.

20. Add 3 μL of 0.5 M iodoacetamide (15 mM) and incubate for 20 min at room temperature (20°C–25°C) in dark to alkylate protein sulfhydryl functional groups.
21. Add 0.5 μL 1 M DTT and incubate 5 min at room temperature (20°C–25°C) to quench the alkylation reaction.
22. Add 2 μL of 50 mM phosphatase inhibitors mix (1 mM each).
23. Add 4 μL of Sequencing trypsin solution (0.5 μg) and incubate the samples overnight (12–16 h) at 37°C.

***Note:*** In case of analyzing pooled fractions, do not increase the trypsin according to the sample volume. Indicated amount (0.5 μg) is sufficient to digest the proteins from the TAP experiment. Use bacteriological incubator or dry block heater with heated lid to avoid liquid condensation at the top of the sample tube.

24. Stop the trypsin reaction by adding 5 μL of 10% TFA (0.5%).

### Peptide desalting using C18 reversed phase chromatography

**Timing: 3 h**

Prepared peptide mixture contains inorganic contaminants from the buffers used in protein elution and protein alkylation that interfere with mass spectrometry analysis. Desalting step is based on peptide capture on C18 column while other buffer components remain in the passing mobile phase. Peptide elution is performed by volatile solution with increased concentration of organic solvent that can be completely evaporated. There are several commercially available peptide desalting kits, such as Thermo Scientific™ Pierce™ C18 Spin Columns, Ultra-Micro SpinColumns (Harvard Apparatus) and C18 Spin Columns (G-Biosciences) with corresponding clean-up protocols for each of them. Here we describe preparation of the C18 desalting microcolumns and their usage for peptide desalting.

25. Insert a frit (approx. 1mm x 1 mm) into 200 μL pipetting tip. Push it gently to final place by a paper-clip wire. The frit can originate from, e.g., a narrow aerosol filter used in 10 μL filter tips. Shorten the 200 μL column by cutting the tip below the frit.
26. Take 2 mL screw cap tube and make a round hole in the cap to accommodate the microtip column.
27. Place the column into the tube. When inserting the microtip column, there should be a space left between the tip and the bottom of the tube to eliminate sample contamination. 2 mL tube will serve as a collection tube for the column conditioning and sample washing.
28. Add approximately 50 μL of 50% C18 resin slurry in acetonitrile (ACN) into the microtip column. Mix well the resin before pipetting.
29. Centrifuge at 300 g for 1 min. The resin column should be 0.5–1.0 cm tall, depending on the microtip geometry. Discard flow-through.
30. Add 100 μL of ACN to condition and settle the column. Centrifuge at 300 g for 1 min.
31. Repeat ACN conditioning step. Discard flow-through.
32. Add 100 μL of 0.1% trifluoroacetic acid (TFA) to equilibrate the column. Centrifuge at 300 g for 1 min.
33. Repeat 0.1% TFA equilibration step. Discard flow-through.
34. Apply the sample to the column. Centrifuge at 300 g for 90 s. Discard flow-through.

***Note:*** In case of larger volumes, apply the sample stepwise in 100 μL aliquots.

35. Add 100 μL of 0.1% TFA to wash the sample. Centrifuge at 300 g for 1 min. Discard flow-through.
36. Repeat 0.1% TFA washing step.
37. Unscrew the tube lid and place it with the microcolumn into Protein LoBind Eppendorf tube.
38. Add 50 μL of TA70 to elute the peptides. Centrifuge at 300 g for 1 min.
39. Repeat the elution step. Pool the eluates.
40. Dry the eluted peptide sample in a vacuum evaporator.

***Note:*** Prepared peptide sample is ready to be dissolved in the sample buffer for proteomic analysis using nano LC-MS/MS technique. Depending on available instruments in individual proteomic facilities, the samples can be analyzed using various nano HPLC systems (e.g., UltiMate 3000 RSLCnano, EASY-nLC) connected to high resolution mass spectrometers of Orbitrap type (e.g. Q Exactive or Exploris series from Thermo Fisher Scientific) or qTOF mass spectrometers (e.g. impactII, timsTOF series from Bruker Daltonics). Obtained MS data can be processed either by corresponding proprietary software tools (Proteome Discoverer, ProteinScape) or by free software packages (MaxQuant, Perseus).

## Expected outcomes

**Figure 2:**
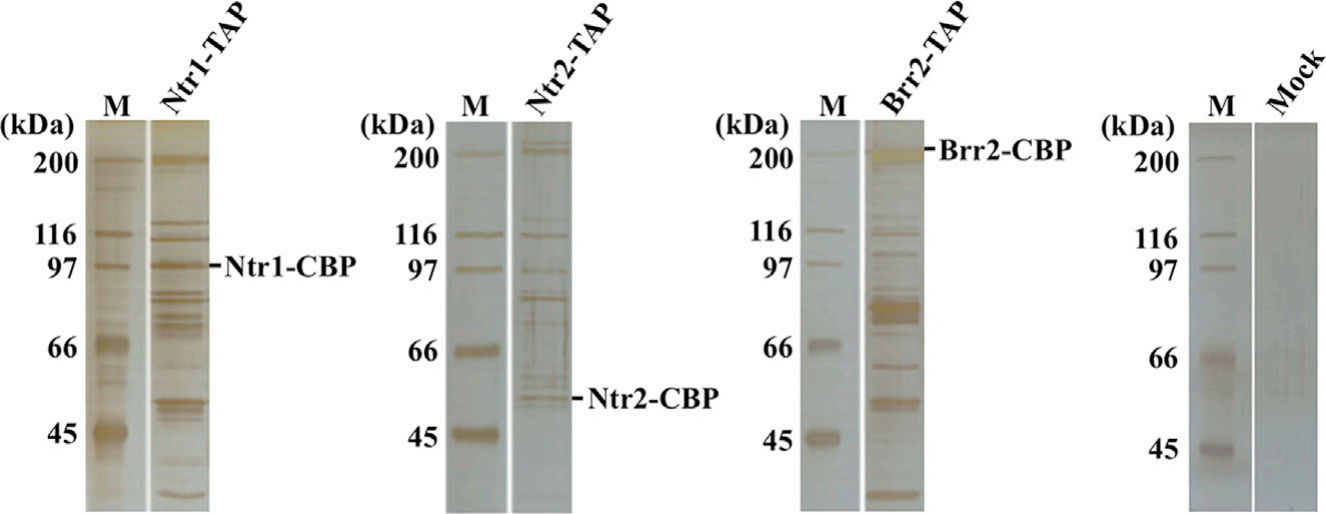
The outcome of tandem affinity purification of TAP-tagged proteins from the fission yeast *S. pombe* TAP-tagged splicing factors Ntr1, Ntr2 and Brr2 were isolated together with co-purifying proteins by tandem affinity purification from *S. pombe* cells. The complexity of isolated complexes was analyzed by 8% SDS-PAGE and proteins were visualized by silver staining. Molecular weight marker (M) is indicated on the left. Positions of Ntr1-CBP, Ntr2-CBP and Brr2-CBP are indicated according to their predicted molecular weights (adapted from Cipakova et al. (2019)).

This protocol is optimized for a large-scale isolation and identification of proteins and their complexes from the fission yeast *S. pombe*. About 30 g of wet cell pellet can be harvested from 6 L of yeast cell culture. Grinding of frozen cell pellets using Freezer/Mill grinder is an efficient way for breaking yeast cells. About 90% of cells are broken when examined by microscopy. Cryomilled cell powder is used to prepare protein extract. Tandem affinity purification allows isolation of TAP-tagged proteins together with co-purifying proteins. Eluted fractions typically contain TAP-tagged protein and its interacting partners but also unspecific protein contaminants. Mass spectrometry analysis can be used to identify purified proteins. Typical results of tandem affinity purified protein complexes are shown in (Figure 2, adapted from (Cipakova et al., 2019)).

## Limitations

This protocol has been successfully used for isolation of several protein complexes. However, this method is unlikely to be optimal for all proteins and alternative purification methods may be needed. A major challenge is to ensure stability of isolated proteins and their complexes during protein extraction and affinity purification. Proteins loosely associated or transiently binding to the TAP-tagged protein may be easily lost during the TAP purification.

There are several limitations concerning the TAP tag. The tag may affect function and/or protein expression levels. To avoid possible artifacts, only functional TAP-tagged constructs should be used. Even if the TAP-tagged protein is functional, it may not be sufficiently exposed to allow binding to the affinity beads. If the TAP-tagged protein is not functional, adding a flexible linker (DNA sequence: gcaggtgctggtgccggagccggtgctggtgct) between the C-terminus of the tagged protein and the TAP tag may help. Although the TEV protease is highly specific, it is useful to keep in mind that the TEV might cleave other proteins that are present in the sample. Finally, it is also possible that the TAP tag is removed from the tagged protein during the purification by cellular proteases.

Another limitation is the need for large volume cultures to collect sufficient amount of yeast cells. In fact, growing and collecting large cultures of yeast cells is time consuming. In this respect, using large volume incubators, centrifuges, filter units and fermenters may be helpful.

Although the TAP protocol minimizes the background, it is likely that non-specific proteins will be present in the final eluate. Comparing samples with untagged control as well as TAP-purifications of unrelated proteins helps to identify contaminants. Some of the common contaminants are listed in Gould et al. (Gould et al., 2004). For less abundant proteins, the signal to noise ratio may be considerably lower and it may be difficult to distinguish between the true complex components and unspecific contaminants.

## Troubleshooting

### Problem 1

The frozen cell pellet is too large to be accommodated into a grinding vial (Prepare yeast cell pellets, Step 8 and Prepare yeast cell powder, Step 9).

### Potential solution

In order to snap-freeze yeast cells in small pieces suitable for filling the grinding vial, place the pelleted cells on ice. Using a spoon cooled in liquid nitrogen, take a small amount of pellet and dip it directly into liquid nitrogen. Repeat until the whole pellet is frozen in a form of small gnocchi. When using the small vial (Cat. #. 6751, sample capacity 0.5–5 g), the size of frozen sample pieces should be approximately 5 mm. For large (Cat#. 6801, sample capacity 5–100 g) and mid-size vials (Cat#. 6881, sample capacity 5–50 g), the frozen pieces should be no larger than 15 mm. Be careful, as larger sample pieces can deflect the impactor sideways into the wall of the cylinder or wedge the impactor against the wall of the cylinder causing breakage. Alternatively, the pellet of collected cells can be placed into a clean plastic bag, frozen at −80°C as a 2–4 mm wide pancake and broken to smaller pieces immediately before filling an appropriate grinding vial.

### Problem 2

The TAP-tagged protein is present at low levels but there is no apparent degradation during the TAP purification (Protein extract preparation and Tandem affinity purification, Steps 1–18).

### Potential solution

Collect and process larger amounts of *S. pombe* cells. For low abundant proteins, we collect 80–100 g of wet cell pellet (20 L of yeast cell culture of OD_600_=0.8–1.0).

### Problem 3

TEV protease cleavage of the TAP-tagged protein is inefficient (Tandem affinity purification, Step 10).

### Potential solution

Some TAP-tagged proteins are not efficiently cleaved during the TEV cleavage step at 16°C. In this case, it is worth trying to perform the TEV cleavage step at higher temperature (e.g., 25°C) for a shorter time. However, special care should be taken to make sure no protein degradation occurs. This can be checked by analyzing proteins bound to IgG beads before and after the TEV cleavage step at higher temperature. Alternatively, TEV protease from a different supplier (e.g., TURBO TEV protease) can be used to check its cleavage efficiency for the particular TAP-tagged protein.

### Problem 4

Excessive protein degradation occurs during TEV cleavage at 16°C (Tandem affinity purification, Step 10).

### Potential solution

Perform TEV cleavage at lower temperature (4°C).

### Problem 5

The amount of isolated/eluted proteins is too low for detection by silver staining (Tandem affinity purification, Step 18, Figure 2).

### Potential solution

The eluted fractions can be concentrated by TCA or acetone precipitation. The precipitated proteins need to be resuspended in 1× SDS loading buffer and resolved by the SDS-PAGE electrophoresis.

## Resource availability

### Lead contact

Further information and requests for resources and reagents should be directed to and will be fulfilled by the lead contact, Juraj Gregan (juraj.gregan@univie.ac.at).

### Materials availability

This protocol did not generate new materials.

## Data Availability

This study did not generate any datasets.
